# Charting the state of GEMs in microalgae: progress, challenges, and innovations

**DOI:** 10.3389/fpls.2025.1614397

**Published:** 2025-06-13

**Authors:** Jacob Tamburro, Nanette R. Boyle

**Affiliations:** ^1^ Quantitative Biosciences & Engineering, Colorado School of Mines, Golden, CO, United States; ^2^ Chemical & Biological Engineering, Colorado School of Mines, Golden, CO, United States

**Keywords:** metabolic flux, algae, photosynthesis, modeling, metabolism

## Abstract

Genome-scale metabolic models (GEMs) provide a systems-level framework for understanding and engineering microalgal metabolism. This review explores the evolution of GEMs in microalgae, highlighting advances in light modeling, automation, and multi-omics integration. Special emphasis is placed on *Chlamydomonas reinhardtii* as a model species. Limitations of current models, particularly for microalgae, are discussed, alongside promising developments in dynamic modeling and machine learning. Together, these innovations chart a path toward more predictive, adaptable GEMs that can accelerate biotechnological applications of microalgae in sustainable production systems.

## Introduction

Microalgae have demonstrated significant potential for the sustainable production of biofuels and other valuable products. As cell factories, microalgae can be optimized for biofuel production (Makareviciene and Sendzikiene, 2022), wastewater processing (Ahmed et al., 2022), and the creation of a wide variety of high-value bioproducts such as nutraceuticals and pharmaceuticals (Abu-Ghosh et al., 2021; Khanra et al., 2022) (see **Table 1**). Microalgae have also been shown to have a solar conversion efficiency of 4.4% (Huntley and Redalje, 2007), considerably higher than the solar conversion efficiency of terrestrial plants which is typically between 1-2% (Vasudevan and Briggs, 2008). The advantage in solar conversion efficiency for microalgae then translates to higher growth rates and annual yields compared to terrestrial plants (Chung et al., 2011). Since many bioproducts produced by microalgae are intracellular, their yields are closely tied to biomass accumulation, meaning that higher growth rates generally result in greater overall production of desired bioproducts.

**Table 1:** Algae species that currently have GEMs reconstructed for them as well as research and cell factory applications of each species.

Species	Research and cell factory metabolite production
*Auxenochlorella protothecoides*	Triacylglycerols (TAGs) overproduction for biofuel (Patel et al., 2018); nutraceuticals: lutein, zeaxanthin (Xiao et al., 2018) and β-Carotene (Park et al., 2018); pharmaceutical: Antibacterial metabolite production (Polat et al., 2023)
*Chlamydomonas reinhardtii*	Model for photosynthesis in microalgae (Harris, 2001); biofuel: overproduction of TAGs (Pandey et al., 2023); biohydrogen production (Kruse et al., 2005); nutraceuticals: lutein, β-Carotene (Rathod et al., 2020), zeaxanthin (Song et al., 2020) and astaxanthin (Ryu et al., 2024), Omega-3 fatty acids (Masi et al., 2023); pharmaceutical: vaccine antigen proteins (Demurtas et al., 2013), camelid heavy chain-only antibodies (Barrera et al., 2015)
*Chlorella variabilis*	Wastewater remediation (Tran et al., 2020); Nutraceutical: lutein (Loganathan et al., 2020), and biofuel: overproduction of TAGs (Sati et al., 2021)
*Chlorella vulgaris*	Nutraceutical: vitamin D, vitamin B12 (Bito et al., 2020), lutein, β-Carotene, Zeaxanthin (Serra et al., 2021) and astaxanthin (Kendirlioglu and Cetin, 2017); biofuel: overproduction of TAGs (Moradi and Saidi, 2022)
*Chromochloris zofingiensis*	Nutraceutical: lutein, zeaxanthin, β-Carotene (Huang et al., 2018) and astaxanthin (Zhang et al., 2021); biofuel: overproduction of TAGs (Vitali et al., 2023)
*Dunaliella salina*	Water remediation (Moayedi et al., 2019; Santos et al., 2001); high salinity tolerance (Hu et al., 2024); nutraceuticals: lutein (Fu et al., 2014), zeaxanthin (Jin et al., 2003), β-Carotene (Xi et al., 2022) and astaxanthin (Chen et al., 2024)
*Emiliania huxleyi*	Broad salinity tolerance (Sheward et al., 2024); nutraceuticals: lutein, fucoxanthin (Zhang et al., 2023), Omega-3 fatty acids (Aveiro et al., 2020)
*Fragilariopsis cylindrus*	Cold tolerant extremophile (Bayer-Giraldi et al., 2010) and nutraceuticals: β-Carotene (Guérin et al., 2024), Fucoxanthin, diadinoxanthin (Guerin et al., 2022) and omega-3 fatty acids (Vaezi et al., 2013)
*Haematococcus pluvialis*	Nutraceuticals: lutein, zeaxanthin, β-Carotene and astaxanthin (Mularczyk et al., 2020); biofuel: overproduction of TAGs (Hosseini et al., 2020)
*Isochrysis galbana*	Broad salinity tolerance (Alkhamis and Qin, 2013); wastewater remediation (Wang et al., 2021); nutraceuticals: fucoxanthin, zeaxanthin, β-Carotene (Chen et al., 2022) and omega-3 fatty acids (Wang et al., 2022); biofuel: overproduction of TAGs (Sánchez et al., 2013)
*Nannochloropsis gaditana*	Nutraceuticals: violaxanthin, Zeaxanthin, β-Carotene (Di Lena et al., 2019) omega-3 fatty acids (Mitra et al., 2015); biofuel: lipid production (Perin et al., 2015)
*Nannochloropsis salina*	Nutraceuticals: violaxanthin (Park et al., 2021), β-Carotene (Brown, 1987) and omega-3 fatty acids (Koh et al., 2024b); biofuel: overproduction of TAGs (Fakhry and El Maghraby, 2015; Koh et al., 2024a)
*Phaeodactylum tricornutum*	Model diatom (Butler et al., 2020); nutraceutical: chrysolaminarin, fucoxanthin, lupeol, botulin and omega-3 fatty acids (Butler et al., 2020); biofuel: overproduction of TAGs (Butler et al., 2020)
*Scenedesmus obliquus*	Wastewater remediation (Álvarez-Díaz et al., 2015), nutraceuticals: lutein (Ho et al., 2014), Neoxanthin, luteoxanthin, violaxanthin, antheraxanthin, β-Carotene (Maroneze et al., 2019), astaxanthin (Qin et al., 2008) and omega-3 fatty acids (Makulla, 2000); biofuel: lipid production (Yang et al., 2020)
*Schizochytrium limacinum*	Nutraceuticals: astaxanthin, canthaxanthin, lycopene, β-Carotene (Zhang et al., 2017), omega-3 fatty acids (Bouras et al., 2020); biofuel: lipid production (Bi et al., 2015)
*Thalassiosira pseudonana*	First microalgae sequenced (Armbrust et al., 2004); wastewater remediation (Wang et al., 2021); nutraceuticals: β-Carotene, fucoxanthin and omega-3 fatty acids (Peng et al., 2024); biofuel: overproduction of TAGs (El-Sheekh et al., 2024)

Unfortunately, algae have not fully realized their potential as cellular factories due to a number of challenges associated with economical production at large scale (Bošnjaković and Sinaga, 2020). A main driver of the overall cost of production is the productivity of the algae (growth rate x production rate) which influences the choice of photobioreactors, separation and labor costs (Acién et al., 2012; Awasthi et al., 2020; Stichnothe et al., 2016). Maximizing productivity can lead to lower downstream costs, and one tool that has been proven to be successful in rerouting carbon in metabolism is metabolic engineering, specifically the use of metabolic models, to predict and implement genetic changes that can improve overall productivity (Hu et al., 2023) and product specific productivity (Yan et al., 2019; Song et al., 2020). For example, metabolic models have been used to guide the overexpression of acetyl-CoA carboxylase to increase lipid accumulation for biodiesel production (Yan et al., 2019) and redirect carbon flux toward carotenoid biosynthesis by optimizing the isoprenoid pathway (Song et al., 2020). These efforts demonstrate how metabolic models can enable precise identification of limiting steps in target pathways and support design strategies to improve yields of economically valuable compounds.

Computational tools provide powerful means to investigate the complexities of metabolism. Among the computational methods employed in metabolic engineering, genome-scale metabolic models (GEMs) stand out due to their relative ease of implementation and comprehensive, systems level approach. GEMs are *in silico* representations of an organism’s metabolic capacity based on the organism’s sequenced genome, enumerating all reactions and metabolites encoded within. Experimental data, such as carbon uptake and excretion, biomass composition and growth rate can be used to constrain the model (Bernstein et al., 2021). Dramatically decreasing costs for high quality genome sequencing has led to increased sequence data for GEM reconstruction (Pareek et al., 2011), and advances in genome annotation have enabled more complete simulations of metabolic processes. GEMs can be used to identify gene knockouts that lead to increased yield or productivity. They can also be used to predict changes in yield due to the incorporation of heterologous metabolic pathways, narrowing the potential mutants to be screened in the lab and drastically decreasing research and development investment (Mekanik et al., 2023). By representing the entire metabolic capacity of an organism, GEMs have also been used to identify genetic targets that are not easy to predict *a priori* as having an impact on the productivity of a specific product (Levering et al., 2016; Yang et al., 2018). The utilization of GEMs is not limited to screening genetic changes, GEMs can additionally be applied to understand how an organism will respond to environmental changes. These applications include media optimization and predictions on the most crucial nutrients for growth (Van Tol and Armbrust, 2021). GEMs also can be utilized to rapidly provide predictions on the changes that varying growth conditions will have phenotypically (Zuniga et al., 2016). *In silico* studies provide an effective method to aid in target selection for traditional experiments, enabling researchers to investigate the impact of thousands of genetic or environmental changes in a fraction of the time it takes to create and characterize in the lab (Ofaim et al., 2021; Nocon et al., 2014).

GEMs have been extensively employed to study metabolism across a wide range of organisms, with the majority of existing literature and models focused on heterotrophic systems such as bacteria and yeast (Orth et al., 2011; Monk et al., 2017; Lu et al., 2019). This emphasis is reflected in the greater availability of heterotrophic GEMs on public repositories such as BioModels (Malik-Sheriff et al., 2020) and BiGG Models (Norsigian et al., 2020). Although some algal GEMs are hosted on these platforms, the listings are not comprehensive and often require manual literature searches to identify additional models. Nonetheless, GEMs in both heterotrophic and autotrophic organisms have proven highly effective for simulating metabolic fluxes, identifying genetic engineering targets and optimizing growth conditions.

Applying GEMs to photoautotrophic organisms, particularly eukaryotic microalgae, presents a distinct set of challenges. These include the need to simulate light-dependent metabolism, diel cycling, and shifting cellular objectives across changing environmental conditions, all within a framework that traditionally assumes steady-state behavior. In this review, we examine the specific difficulties encountered when constructing and utilizing GEMs for photoautotrophic microalgae, as well as the current limitations that hinder their broader adoption and predictive accuracy. A dedicated section explores the role of *Chlamydomonas reinhardtii*, which has emerged as a cornerstone species in algal systems biology and a model for developing and refining GEMs in microalgae. Finally, we highlight future directions in GEM research, including the integration of dynamic modeling, multi-omics data, and machine learning techniques, all of which are poised to significantly advance the utility of GEMs in both fundamental research and applied biotechnology.

## *Chlamydomonas reinhardtii*: a keystone species for microalga GEM reconstruction

**Figure 1:**
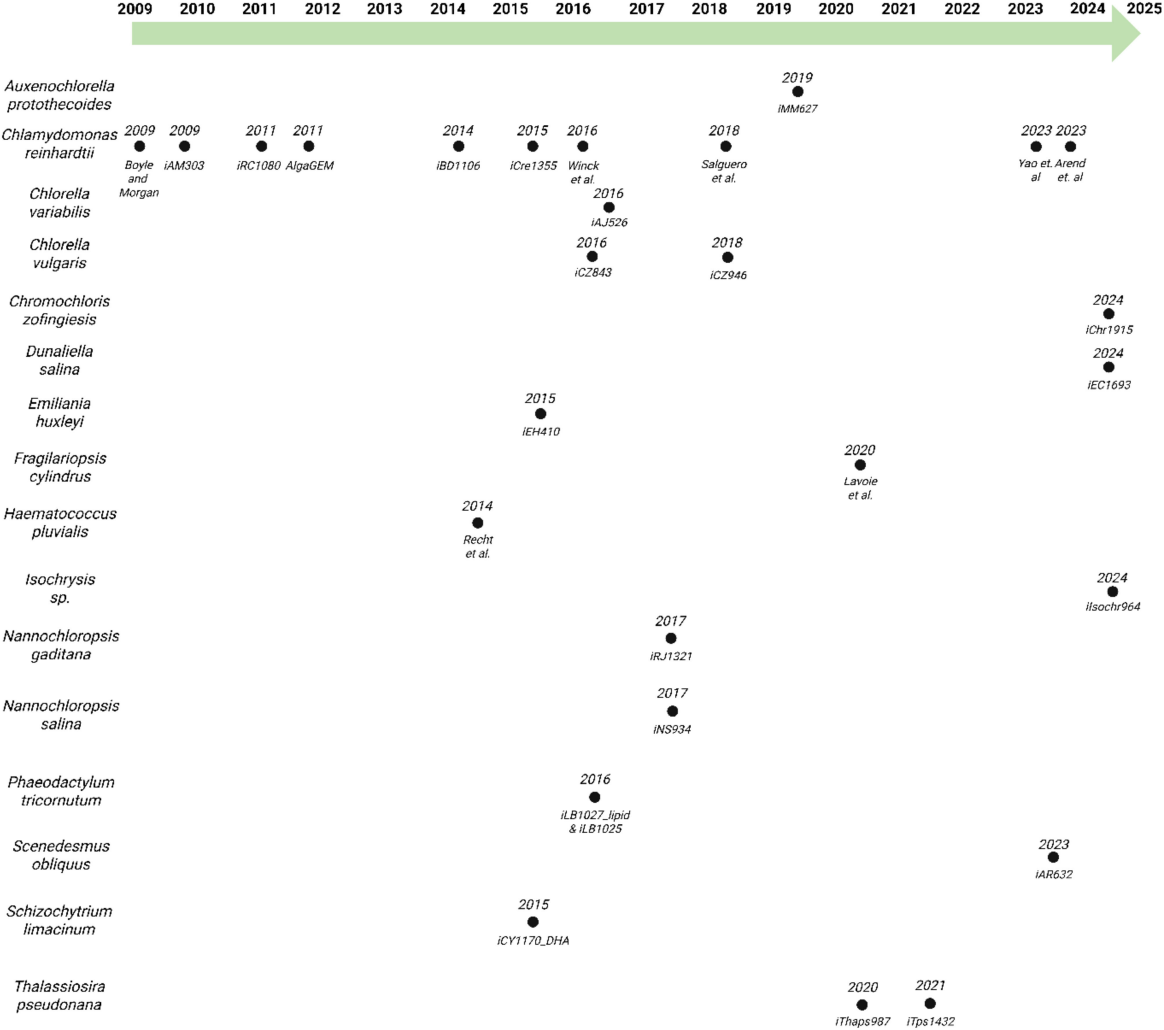
Historical perspective on the generation of algal GEMs, organized by species and year of publication.

*Chlamydomonas reinhardtii* has received extensive attention in scientific research (Harris, 2001), emerging as a pivotal organism for studying microalgae and aquatic photosynthetic systems (Calatrava et al., 2023). As a model green microalga, *C. reinhardtii* has served as the foundation for GEMs in algal species. The first GEM for *C. reinhardtii* was developed by Boyle and Morgan in 2009 (Boyle and Morgan, 2009), marking the first GEM constructed for any algal species (see **Figure 1**). Another noteworthy GEM is *iCre1355* (Imam et al., 2015), which has served as a foundational platform for subsequent models. Derived like many of the currently available GEMs from the earlier *iRC1080* (Chang et al., 2011), *iCre1355* (Imam et al., 2015) incorporates updates based on improvements made to the annotation of the genome, rectifying inaccuracies in gene-protein reaction associations. This improved model has been utilized to predict growth under varying light conditions (Shene et al., 2018). *iCre1355* (Imam et al., 2015) was also utilized in the development of the first diurnal metabolic model in microalgae developed by Metcalf and Boyle (Metcalf Alex and Boyle Nanette, 2022). This diurnal model is a type of transient metabolic model (TMM). TMMs are computational models that capture dynamic changes in metabolism under varying environmental conditions. The Metcalf and Boyle TMM incorporated quantitative, time dependent transcriptomic data to constrain the availability of the associated gene products and metabolic reactions and more accurately predict growth in diurnal conditions.

The GEM developed by Yao et al. (2023), merged the *iCre1355* and *iGR774* models, replacing the chloroplast reactions in *iCre1355* (Imam et al., 2015) with the more detailed *iGR774* (Bjerkelund Rokke et al., 2020) chloroplast specific model. This integration allowed for a more biologically accurate depiction of chloroplast metabolism, improving compartmental resolution, gene-reaction mapping, and the model’s ability to simulate light-driven and plastid-localized processes. Yao et al. additionally utilized protein constrained flux balance analysis (PC-FBA), an extension of traditional FBA that integrates enzyme capacity and proteome allocation to better reflect cellular limitations. This approach allows for context-specific flux predictions informed by transcriptomic data and represents the first implementation of a protein-constrained model (PC-Model) for a microalgal GEM.

More recently, Arend et al (Arend et al., 2023). continued this advancement by directly integrating quantitative proteomic data to constrain enzyme usage, offering a more accurate representation of *in vivo* metabolic states. This proteomics-driven approach narrows the solution space of the model, leading to improved predictions of enzyme allocation and flux distributions. With these advancements, *C. reinhardtii*’s GEMs continue to be at the forefront of advancing algal biotechnology, significantly contributing to the understanding of microalgal metabolism and algal GEM reconstructions.

## Challenges and limitations of algal genome-scale metabolic models

GEMs are a powerful and rapidly advancing tool for understanding cellular metabolism, however, like any complex modeling approach, there are challenges that researchers continue to address to unlock their full potential. One challenge across all organisms but particularly in non-model species is inaccurate or incomplete genome annotations, which leads to gaps that need to be manually filled. This issue is particularly pronounced in photoautotrophic organisms such as microalge, as fewer well-annotated reference genomes are available for comparison. *C. reinhardtii* largely represents an exception as its genome has undergone extensive sequencing and curation (Craig et al., 2023), as well as support by databases such as Phytozome (Goodstein et al., 2012), ChlamyCyc (May et al., 2009) and AlgePath (Zheng et al., 2014). Not all organisms have this extensive research with many inaccuracies arising from the need for homology-based annotations, which while faster than manual curation, can assign functions without biochemical validation. Based on how automated annotation algorithms work, poor annotations can be carried through to new organisms. An additional challenge with annotation is that many metabolic pathways and reactions, particularly in non-model organisms, are still being discovered or refined, which can create gaps in the models that require extensive manual curation or assumptions to fill (Karp et al., 2018). Beyond annotation issues, GEMs also face limitations due to their reliance on stoichiometric reactions rather than reaction kinetics. By ignoring reaction kinetics, the entire metabolic network can be modeled; but it comes at a cost because the level of detail is greatly reduced. Kinetic models have been developed for well-studied organisms such as *Escherichia coli* (Khodayari et al., 2014), but they include far fewer reactions than GEMs due to the requirement for detailed kinetic data. For microalgae, such data is especially scarce, making GEMs the most practical framework for modeling their metabolism. To enhance their accuracy, GEMs can integrate omics data such as transcriptomics and proteomics. This data provides crucial insights into cellular states and responses. However, aligning diverse omics datasets with GEMs is another challenge, requiring sophisticated computational techniques. Fortunately, advancements in data integration and computational methods are allowing GEMs to incorporate omics data more effectively and enhance their predictive power (Sen and Orešič, 2023). However, even with these advancements in annotation and omics integration, GEMs still face limitations due to key assumptions most notably the reliance on steady state conditions that pose unique challenges in photosynthetic organisms.

Adopting a steady-state assumption poses significant challenges for GEMs in photosynthetic microalgae, where complex diel fluctuations and regulatory mechanisms make strict steady-state models less representative of metabolic dynamics. While this assumption is important mathematically, converting a set of ordinary differential equations to a set of linear equations, it limits the application of GEMs to steady growth conditions. This is particularly pronounced in photosynthetic organisms due to typical growth in diel light conditions which results in substantial fluctuations in metabolism (Fisher et al., 2023) due to the shift from day to night and vice versa. Photosynthesis also involves numerous regulatory mechanisms, such as photoprotection (Goss and Jakob, 2010), photosynthetic quenching (Schubert et al., 2006), and variations in photon flux (Schnurr et al., 2016), all of which are difficult to represent with static models. To account for regulatory elements such as enzyme capacity constraints and gene expression control, the integration of proteomic and transcriptomic data into GEMs is essential. Transcriptomics can be used to infer active pathways by adjusting reaction constraints based on gene expression levels, while proteomics enables more accurate estimation of enzyme abundances and capacities to constrain the solution space of the model. However, such genome-wide data sets remain scarce for most microalgae due to limited experimental and financial investment. *Chlamydomonas reinhardtii* stands out in this regard, as it benefits from available transcriptomic and proteomic data. An extension of this problem is the use of a single objective function (most often to maximize biomass). While this objective function matches the cellular objective for heterotrophic bacteria quite well (Orth et al., 2011), this objective is especially problematic in photosynthetic organisms due to the decoupling of carbon and energy inputs and the time-dependent nature of cellular division in diel light. Additionally, the biomass function for algae is more complex and dynamic than those seen in heterotrophic organisms, as many can grow in autotrophic, mixotrophic, and heterotrophic states. Each of these trophic states requires a distinct biomass formulation to reflect the underlying physiological differences (Matos et al., 2017). Moreover, algal cells must continuously optimize their metabolism in response to environmental conditions. These can vary such as minimizing energy usage when light is not present or the formation of storage products in preparation for environmental changes. Additionally autotrophic and mixotrophic growth results in biomass composition are more dependent on the environment, changing with light intensity throughout the day under diel conditions (Jallet et al., 2016).

**Figure 2:**
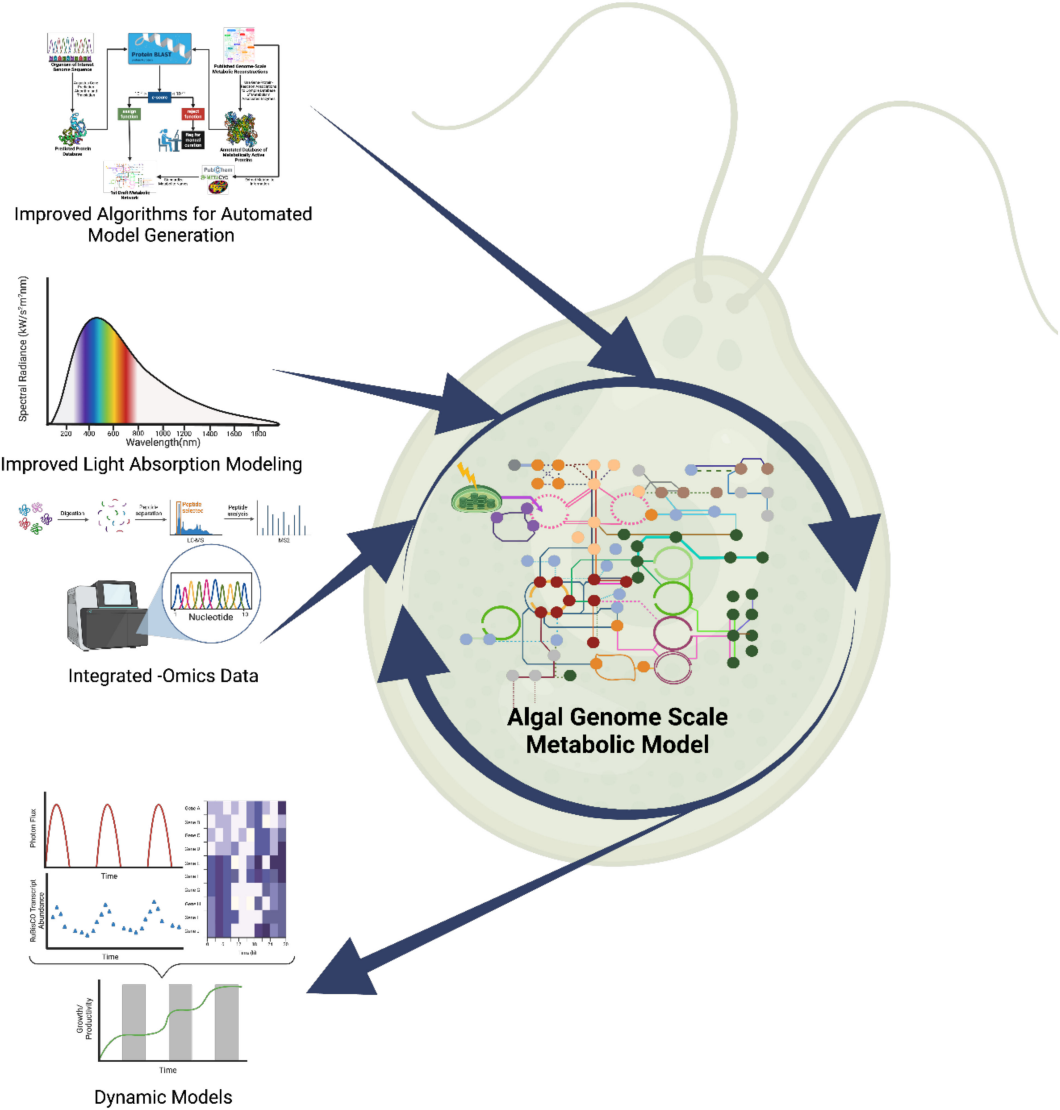
Challenges for algal GEM reconstruction include the need for automated algorithms for model reconstruction specifically for algae, improved light modeling and integrating -omics data to improve predictability. Addressing these will enable the design of dynamic models that can better predict growth in dynamic conditions, such as day/night cycles.

These challenges have motivated the development of more sophisticated GEMs that better capture the complexity of photosynthetic microalgae. Recent models have begun to incorporate multiple objective functions, simulate compartmentalized metabolism and account for trophic flexibility. Other innovations address environmental responsiveness, such as stress adaptation and diel regulation. The following sections highlight these advancements through examples of automated reconstruction tools, light modeling, omics integration, and dynamic modeling (see **Figure 2**).

## Automation of model reconstruction

Automated reconstruction of GEMs helps address the time-intensive nature of model development by streamlining the reconstruction process, making it feasible to generate high-quality models for a wider range of organisms. The GEM *iChr1915* (Meagher et al., 2024) for *Chromochloris zofingiensis* represent significant advancement in the automatic curation of photosynthetic metabolic networks. *iChr1915* (Meagher et al., 2024) utilized an algorithm called Rapid Annotation of Photosynthetic Systems (RAPS) (Metcalf et al., 2020) to automate much of the process. Other GEM automation tools exist such as model SEED (Devoid et al., 2013) and CarveMe (MaChado et al., 2018), however these automation tools are not tailored for use on algae. The model SEED (Devoid et al., 2013) framework plantSEED (Seaver et al., 2014) is, as its name would suggest, better suited for the reconstruction of plant GEMs as it carries over many highly conserved reactions in plants to avoid issues with gap filling. Including these conserved reactions in algal GEM reconstructions doesn’t properly represent the diversity of microalgal metabolism (Catalanotti et al., 2013) and variation from plant metabolism (Tamoi and Shigeoka, 2015). CarveMe (MaChado et al., 2018) additionally is primarily for the reconstruction of prokaryotes and bacterial communities with reactions pulled from the BiGG database (Schellenberger et al., 2010) excluding reactions unique to eukaryotic organisms. The use of RAPS (Metcalf et al., 2020) enabled the development of a high quality first draft network in only 20 minutes; the resulting model only required minimal manual curation. RAPS (Metcalf et al., 2020) facilitates the automated curation of GEMs for photosynthetic algae by leveraging manual curation efforts already invested in published models and using these to generate new models.

Another automation tool that has been utilized in GEM reconstruction is RAVEN toolbox (Agren et al., 2013), which was utilized in the reconstruction of *iLB1027_lipid* and *iLB1025 (* Levering et al., 2016 *).* The original RAVEN toolbox provided a MATLAB-based framework to facilitate semi-automated draft reconstruction of metabolic networks through homology-based mapping from annotated genomes to template models. In this case, RAVEN was used to generate an initial draft network by identifying homologous genes based on previously published models from photosynthetic organisms. This draft network served as the foundation, which was further refined using updated genome annotations, subcellular localization predictions, and biochemical validation. Although significant manual effort was required to correct compartmentalization, balance reactions, and incorporate complex eukaryotic features, the automated steps provided by RAVEN accelerated the initial reconstruction process and ensured alignment with known gene–reaction relationships. A newer version of the RAVEN toolbox, RAVEN 2.0 (Wang et al., 2018), has since been developed with expanded capabilities, including integration of MetaCyc-based reconstruction (Caspi et al., 2020) and improved model interoperability.

These method addresses a key challenge in GEM development: the time-consuming nature of manual curation and annotation gaps. By using RAPS and RAVEN, researchers can streamline the initial stages of model development, allowing them to focus on gap-filling and other manual curation efforts that will lead to a high-quality network. This hybrid approach reduces the time-intensive nature of fully manual curation by automating the initial draft creation and filling metabolic gaps while still incorporating the precision of expert intervention where needed.

## Modeling light harvesting

Because microalgae are photosynthetic organisms incorporating light dynamics such as wavelength, intensity and spectral composition into GEMs is crucial for accurately capturing their metabolism and improving model predictions. The first GEM for microalgae to account for different wavelengths of photons in its metabolic network was *iRC1080* (Chang et al., 2011), a model for *C. reinhardtii* allowing for variations in light conditions to influence the model. *iRC1080* (Chang et al., 2011) achieved this by defining spectral ranges associated with all the photon-utilizing reactions in the network connecting and allowing for 11 distinct light sources such as solar light as well as halogen and LED lights to be modeled. The metabolic network was also verified with over 90% of transcripts predicted by *iRC1080* (Chang et al., 2011) being found in experimental transcriptomic data. Additionally, *iRC1080* (Chang et al., 2011) accurately predicted solar conversion efficiency to be 2%, matching experimental results. The coupling of light wavelengths with reactions marked a substantial improvement on previous models and allows for the optimization of light sources as well as elucidating the phenotypic results of varying light conditions. Similarly, the *Chlorella variabilis* model *iAJ526* (Juneja et al., 2016) accounts for varying light conditions by simulating the effects of twelve different light sources on growth rate and uptake rates. These light sources were like those modeled in iRC1080 (Chang et al., 2011) representing light sources that have been utilized in algal growth, but had a greater focus on modeling different combinations of LED light and didn’t include sunlight. Three of these light conditions were experimentally validated, confirming predictions made by the model that white light would provide the best growth followed by red/blue light then red light. *iAJ526* (Juneja et al., 2016) predicts higher growth rates than those observed experimentally under all light conditions with the authors attributing the differences to issues with the model’s lack of growth kinetics and photoinhibition. These models advance GEM reconstruction in algae and other photosynthetic organisms by offering a more robust representation of the effects light intensity and composition have on metabolism.

Innovations in GEMs for microalgae have also addressed other limitations traditionally seen in GEMs, particularly those affecting photosynthetic organisms. For instance, the *Thalassiosira pseudonana* model *iTps1432* (Van Tol and Armbrust, 2021) incorporates the application of photon loss reactions to simulate photosynthetic quenching. By including these reactions, *iTps1432* (van Tol and Armbrust, 2021) offers valuable insight into photon loss reactions with particular interest coming from predictions around cyclic electron flow at low light intensities. At these lower light intensities, the model predicts that a significant portion of total electron flow is made up of cyclic electron flow supporting other findings highlighted in the paper that cyclic electron flow is important for ATP generation at low light (Bailleul et al., 2015). Cyclic electron flow is not only important for ATP generation and modeling light dynamics but has also been demonstrated to be important in lipid biosynthesis pathways in algae (Chen et al., 2015). This highlights the potential improvements adding light dynamics reaction within GEMs can provide. With this added insight these models can be better applied to determine targets for improving metabolic engineering outcomes under autotrophic conditions.

## Models with a focus on reactions outside of carbon metabolism

GEMs can be applied to explore algal production of value-added compounds beyond traditional targets like biomass and hydrocarbons. One such emerging application is the modeling of green hydrogen production, which has gained significant interest in recent years (Borges et al., 2024). Models such as *iMM627* (Mekanik et al., 2019) for *Auxenochlorella protothecoides* and *iRJ1321* (Shah et al., 2017) for *Nannochloropsis gaditana* incorporate predictions of hydrogen production. The *iMM627* (Mekanik et al., 2019) model integrates two objective functions maximizing both biomass and hydrogen production. By incorporating multiple objectives, the model can more completely utilize the GEMs metabolic network and better represent reactions outside of central carbon metabolism. Additionally, although not originally designed for hydrogen production, the AlgaGEM model (Gomes De Oliveira Dal’molin et al., 2011) for *Chlamydomonas reinhardtii* was used to maximize hydrogen synthesis through modification of its objective function, demonstrating that any genome-scale metabolic model can, in principle, be adapted to study hydrogen production or any other product based on the set objective function.

Beyond expanding product scope, recent GEMs have also improved pathway resolution for key metabolic processes such as nitrogen metabolism. The *Nannochloropsis salina* model *iNS934* (Loira et al., 2017) provides a more detailed representation of nitrogen metabolism, capturing the intricate balance between carbon fixation and nitrogen assimilation, while also incorporating a variety of nitrogen sources. This allows *iNS934* (Loira et al., 2017) to integrate essential reactions not directly tied to carbon metabolic pathways, addressing gaps present in earlier models and offering more flexibility when optimizing media recipes. Such refinements enhance the model’s utility for strain engineering under nutrient-limited conditions and support the development of cost-effective cultivation strategies.

## Model robustness

Enhancing the robustness of GEMs improves their ability to simulate organismal responses to environmental stress and genetic perturbations, making them more reliable tools for predictive modeling and metabolic engineering. The Lavoie et al. *Fragilariopsis cylindrus* model (Lavoie et al., 2020) focuses on reaction robustness to help analyze how metabolic networks maintain stability under stress or environmental shifts. This robustness analysis, combines flux balance analysis (FBA) with minimization of metabolic adjustment (MOMA) (Segrè et al., 2002) allows for better prediction of how networks respond to perturbations made by knock outs. In contrast to flux variability analysis (FVA) (Gudmundsson and Thiele, 2010), which assesses the flexibility of individual reactions by calculating the range of fluxes consistent with optimal growth, MOMA evaluates robustness based on the assumption that, following a perturbation, the network minimizes its deviation from the wild-type flux distribution without immediately reoptimizing for a new objective. This makes MOMA particularly useful for modeling short-term or acute responses, when the organism has not yet had time to adapt through regulation or evolution. This improves the GEM’s ability to simulate stress responses, addressing a significant aspect of how *F. cylindrus* survives well in its very dynamic environment (Yoshida et al., 2020).

The Recht et al. model (Recht et al., 2014) for *Haematococcus pluvialis* further incorporates variability flux sampling (VFS), an additional step on the commonly used FVA. VFS enables more accurate flux predictions and a deeper analysis of metabolic pathways as it not only predicts the range of possible fluxes, as is done in FVA, but also includes determinations about the probabilities of various fluxes. Incorporating VFS allows for better understanding of pathways that are activated as a stress response as demonstrated in the models focus on exploring the shift toward fatty acid synthesis under nitrogen starvation. Variability Flux Sampling (VFS) enhances interpretation of flux flexibility by generating probability distributions of feasible flux values through random sampling and constrained optimization, rather than assuming a single optimal flux solution. This allows models to reflect the range and likelihood of alternative flux states under given physiological constraints. While not inherently dynamic, the application of VFS across time-resolved datasets such as in *H. pluvialis* under nitrogen deprivation captures experimentally observed shifts in metabolism, including the transition from carbohydrate accumulation to fatty acid biosynthesis (Recht et al., 2012), underscoring the need for models that can represent metabolic plasticity under stress. Although demonstrated here in the context of specific GEMs, VFS and MOMA are generalizable approach that can be applied to any GEM to enhance the characterization of condition-dependent metabolic states.

## Integration of additional omics data and dynamic modeling

Integrating omics data into GEMs enhances their predictive power by capturing regulatory and physiological constraints that are not represented by purely stoichiometrically models. The Yao et al. model (Yao et al., 2023) for *C. reinhardtii* does this by incorporating RNA sequencing data to assume the proteome of the organism as well as enzyme data to create a protein-constrained metabolic model (PC-model). This allows for the model to better represent the dynamics that are lost in the conventional approach of representing metabolism only stoichiometrically. However, while transcriptomics provides useful insights into gene expression, it does not fully reflect metabolic activity due to regulatory layers such as translation, protein turnover, and post-translational modifications. The model by Arend et al (Arend et al., 2023). published shortly after the Yao et al. model advances this framework by directly incorporating quantitative proteomic measurements. The data collected was used to calculate *in vivo* apparent turnover numbers (k_app_) for 568 reactions, providing a more accurate basis for constraining enzyme usage within the model. Of the 1460 enzymes, 936 (64%) were quantified in at least one experimental condition, representing the most extensive proteome coverage achieved for *C. reinhardtii* to date. This allowed the model to more accurately constrain enzyme usage by grounding flux predictions in measured protein abundances, thereby significantly reducing the solution space and increasing the physiological relevance of the predicted flux distributions. By aligning enzyme usage with what is actually present in the cell, the model more faithfully captures metabolic capabilities. Models that incorporate omics data have great potential in better representing the complex regulatory mechanisms present around metabolism (Carthew, 2021) as well as applications under varying growth conditions (Gim et al., 2016).

Another model, *iEH410* (Knies et al., 2015) for *Emiliania huxleyi*, introduces diurnal FBA (diuFBA), significantly improving the simulation of internal regulation of metabolic reactions by moving beyond static flux distributions and better reflecting real-time cellular responses. diuFBA simulates the organism’s metabolism under alternating light and dark conditions. This approach partitions a 24-hour diurnal cycle into discrete light and dark phases, assuming quasi-steady-state conditions within each phase. Another important feature of this model is that it allows for dynamic optimization of storage metabolites, such as mannitol and lipids, rather than relying on fixed concentrations set by the biomass function, as is standard. To achieve this dynamic optimization, diuFBA extends the stoichiometric matrix to include duplicated networks for the light and dark periods, which are connected through reversible transfer reactions for storage metabolites. The model integrates fluxes over each phase duration using explicit Euler integration, enabling the calculation of net concentration changes across the full cycle. This formulation preserves the structure of classical FBA, allowing for efficient convex optimization while capturing the temporal redistribution of metabolic resources that occurs in response to circadian environmental changes. By solving for metabolite accumulation across light and dark periods within a single optimization problem, diuFBA offers a more biologically relevant representation of photosynthetic metabolism without the computational complexity of fully dynamic simulations. However, while this approach captures resource allocation across day-night transitions, it still assumes steady-state behavior within each phase and cannot represent short-term metabolic fluctuations. This limitation has motivated the development of transient metabolic models (TMMs), which aim to simulate cellular metabolism at finer temporal resolution under continuously changing environmental conditions.

TMMs offers a promising avenue for future research utilizing the value of GEMs while offering a dynamic model. While dynamic models have been developed for heterotrophic organisms such as *E. coli* (Yang et al., 2019) and photosynthetic species like *Synechocystis* sp (Rügen et al., 2015), similar models have been largely absent in microalgae. The first TMM for microalgae was developed by Metcalf and Boyle (Metcalf Alex and Boyle Nanette, 2022) in *C. reinhardtii* to model growth in diel light. The model was based on experimental transcriptomics data based on growth in 12:12 hour day:night cycles as this data was used to constrain the availability of the associated enzymatic reactions based on gene expression data. Additionally, the TMM also decoupled the biomass objective functions from the standard static biomass equation allowing it to better simulate the cells adapting to the changing environmental conditions over a day. This is a substantial improvement on GEMs, addressing one of their key challenges: that they are generally static stoichiometric representations of metabolism. The dynamics of the TMM also allow for better targeting for metabolic engineering as these models better represents the fluctuations in metabolism over the course of a day rather than at a single point in the day. Despite these advantages, the implementation of TMMs depends on high-resolution, time-series transcriptomic data, the generation and integration of which are both labor-intensive and expensive. However, the ability to simulate time-resolved shifts in gene expression and metabolism makes this investment particularly valuable, especially for photosynthetic organisms where diel dynamics are fundamental to metabolic function.

## Prospects for future microalgal genome-scale metabolic models

Future advances in GEM formulation will enable more sophisticated models that will be better suited to predicting the dynamic and complex metabolism of microalgae. While GEMs have traditionally relied on steady-state assumptions using FBA, incorporating regulatory constraints has successfully been demonstrated in the Yao et al. model and *iEH410*. While both these GEMs incorporated transcriptomics data, there are further advancements that can be made to the reconstruction of future GEMs incorporating multi-omics data. Tools such as GECKO 2.0 (Domenzain et al., 2022) allow for pipelines for the implementation of enzyme kinetic parameters and proteomic data into GEMs which has already been utilized in multiple species of yeast, *E. coli* and *Homo sapiens*. By adding additional layers of omics data GEMs can address limitations that are presented in many of the currently available static stoichiometric models.

Another emerging direction for algal GEMs is the application of microbial community models (MCMs), which have garnered considerable interest in recent years (Tarzi et al., 2024). MCMs capture the complex inter-specific interactions that microalgae experience in both natural and engineered environments. Rather than existing in isolation, algae typically coexist with diverse microbial partners that influence their metabolism through nutrient exchange, competition, and metabolic cross-feeding. To simulate these interactions, community-scale modeling tools such as SteadyCom (Chan et al., 2017), MICOM (Diener et al., 2020), and the Microbiome Modeling Toolbox (Heinken and Thiele, 2022) enable constraint-based simulations that consider the growth and resource allocation strategies of multiple interacting species. Building on this, dynamic models including dOptCom (Zomorrodi et al., 2014) and COMETS (Harcombe et al., 2014) incorporate spatial and temporal variation, making them especially suited for studying nutrient shifts and microbial succession. Incorporating algal GEMs into these MCM frameworks could improve predictive accuracy under realistic conditions, uncover emergent properties such as division of labor and metabolite sharing, and support the design of more productive algal–bacterial consortia for biotechnology applications. While data availability remains a constraint for many microalgal species, machine learning offers exciting opportunities. In particular, deep learning, which uses neural networks to perform multi-level predictions (Lecun et al., 2015), has already improved genome annotations in bacterial metagenomes (Boer et al., 2024). Applying similar approaches to microalgae could enable the reconstruction of GEMs for the vast number of algal species that remain unculturable (Sharma and Rai, 2010). This could not only improve our understanding of these organisms but also help design more effective cultivation strategies.

Another promising avenue is the integration of GEMs with Transient Metabolic Models (TMMs), which simulate metabolic changes over time and under varying environmental conditions. While a TMM has been developed for *Chlamydomonas reinhardtii* (Metcalf Alex and Boyle Nanette, 2022), other microalgae including those with GEMs (see **Table 2**) currently lack such dynamic models. Expanding TMMs to include additional species and conditions such as UV radiation, temperature fluctuations, and nutrient availability could dramatically enhance the applicability of GEMs in modeling real-world scenarios (El-Sheekh et al., 2021; Al Jabri et al., 2021; Ikaran et al., 2015).

**Table 2:** The table displays the GEMs currently published, organized by species and year of publication from oldest to newest.

Species	Model	Year	Reactions	Metabolites
*Auxenochlorella protothecoides*	*iMM627* (Mekanik et al., 2019)	2019	1,963	2,115
*Chlamydomonas reinhardtii*	Boyle and Morgan (Boyle and Morgan, 2009)	2009	484	458
	*iAM303* (Manichaikul et al., 2009)	2009	259	267
	*iRC1080* (Chang et al., 2011)	2011	2,190	1,706
	*AlgaGEM* (Gomes De Oliveira Dal’molin et al., 2011)	2011	1,725	1,862
	*iBD1106* (Chaiboonchoe et al., 2014)	2014	2,445	1,959
	*iCre1355* (Imam et al., 2015)	2015	2,394	1,845
	Winck et al (Winck et al., 2016)	2016	3,554	2,342
	Salguero et al (Mora Salguero et al., 2018)	2018	3,726	2,436
	Yao et al (Yao et al., 2023)	2023	2,641	2,240
	Arend et al (Arend et al., 2023)	2023	2,394	1,845
*Chlorella variabilis*	*iAJ526* (Juneja et al., 2016)	2016	1,455	1,236
*Chlorella vulgaris*	*iCZ843* (Zuniga et al., 2016)	2016	2,294	1,770
	*iCZ946* (Zuniga et al., 2018)	2018	2,294	1,770
*Chromochloris zofingiensis*	*iChr1915* (Meagher et al., 2024)	2024	3,413	2,652
*Dunaliella salina*	*iEC1693* (Cunha et al., 2024)	2024	4,614	3,732
*Emiliania huxleyi*	*iEH410* (Knies et al., 2015)	2015	410	363
*Fragilariopsis cylindrus*	Lavoie et al (Lavoie et al., 2020)	2020	2,144	1,707
*Haematococcus pluvialis*	Recht et al (Recht et al., 2014)	2014	2,622	1,975
*Isochrysis* sp.	*iIsochr964 (* Sengupta et al., 2024 *)*	2023	4,315	1,879
*Nannochloropsis gaditana*	*iRJ1321 (* Shah et al., 2017 *)*	2017	1,918	1,862
*Nannochloropsis salina*	*iNS934 (* Loira et al., 2017 *)*	2017	2,345	1,985
*Phaeodactylum tricornutum*	*iLB1027_lipid (* Levering et al., 2016 *)*	2016	4,456	2,172
	*iLB1025 (* Levering et al., 2016 *)*	2016	2,156	1,704
*Scenedesmus obliquus*	*iAR632 (* Ray et al., 2023 *)*	2023	1,476	1,549
*Schizochytrium limacinum*	*iCY1170_DHA (* Ye et al., 2015 *)*	2015	1769	1659
*Thalassiosira pseudonana*	*iThaps987 (* Ahmad et al., 2020 *)*	2020	2,477	2,456
	*iTps1432 (Van Tol and Armbrust, 2021)*	2021	6,073	2,789

Altogether, these innovations including multi-omics integration, machine learning, and dynamic modeling represent the future of microalgal GEMs. They offer a more comprehensive understanding of algal metabolism, particularly under diel cycles and photosynthetic fluctuations, moving the field closer to realizing the full potential of microalgae in biotechnology and sustainability applications.

## Conclusion

Microalgae hold immense potential for contributing to a sustainable future through their applications in biofuels, bioremediation, and the production of high-value products. The development of GEMs has emerged as a powerful tool in understanding the complex metabolic networks of these organisms, enabling researchers to optimize their metabolic pathways effectively. However, while GEMs have made significant strides, they are not without limitations. Issues related to incomplete genome annotations, static assumptions, and the integration of multi-omics data continue to pose challenges for GEMs to more accurately simulate metabolism. To address these limitations and fully harness the capabilities of microalgae, there is a pressing need for the creation of more GEMs across a diverse array of algal species. Expanding the repertoire of GEMs will enhance our understanding of algal metabolism and facilitate the development of tailored strategies for metabolic engineering. By addressing the existing challenges and improving GEM methodologies, we can pave the way for a more environmentally friendly future, ultimately contributing to a more sustainable and productive bioproduct landscape.
